# Changes in cortical activity associated with adaptive behavior during repeated balance perturbation of unpredictable timing

**DOI:** 10.3389/fnbeh.2015.00272

**Published:** 2015-10-14

**Authors:** Andreas Mierau, Thorben Hülsdünker, Heiko K. Strüder

**Affiliations:** Institute of Movement and Neurosciences, German Sport University CologneCologne, Germany

**Keywords:** posture, EEG, N1, P1, adaptation, falls

## Abstract

The compensation for a sudden balance perturbation, unpracticed and unpredictable in timing and magnitude is accompanied by pronounced postural instability that is suggested to be causal to falls. However, subsequent presentations of an identical perturbation are characterized by a marked decrease of the amplitude of postural reactions; a phenomenon called adaptation or habituation. This study aimed to identify cortical characteristics associated with adaptive behavior during repetitive balance perturbations based on single-trial analyses of the P1 and N1 perturbation-evoked potentials. Thirty-seven young men were exposed to ten transient balance perturbations while balancing on the dominant leg. Thirty two-channel electroencephalography (EEG), surface electromyography (EMG) of the ankle plantar flexor muscles and postural sway (i.e., Euclidean distance of the supporting platform) were recorded simultaneously. The P1 and N1 potentials were localized and the amplitude/latency was analyzed trial by trial. The best match sources for P1 and N1 potentials were located in the parietal (Brodmann area (BA) 5) and midline fronto-central cortex (BA 6), respectively. The amplitude and latency of the P1 potential remained unchanged over trials. In contrast, a significant adaptation of the N1 amplitude was observed. Similar adaptation effects were found with regard to postural sway and ankle plantarflexors EMG activity of the non-dominant (free) leg; i.e., an indicator for reduced muscular co-contraction and/or less temporary bipedal stance to regain stability. Significant but weak correlations were found between N1 amplitude and postural sway as well as EMG activity. These results highlight the important role of the midline fronto-central cortex for adaptive behavior associated with balance control.

## Introduction

The compensation for unpredictable balance perturbations to regain postural stability is essential to avoid falls. There is growing evidence suggesting that the cerebral cortex is crucially involved in controlling such compensatory reactions. Specifically, it has been shown that transient balance perturbations are associated with characteristic changes in electroencephalographic (EEG) activity, the so-called perturbation-evoked response (PER).

A disruption of balance initially elicits a positive potential most pronounced over the centro-parietal cortical region after about 40–60 ms, referred to as the P1 response (Grünewald et al., 1984; Dietz et al., 1985a; Ackermann et al., 1986; Duckrow et al., 1999; Quant et al., 2005). The P1 is largely insensitive to contextual changes in perturbation application (Adkin et al., 2006) and its latency is delayed in patients with slow conduction velocity in peripheral nerves (Dietz et al., 1985b). Therefore, the P1 response has been suggested to reflect the initial sensory representation of the perturbation-induced afferent feedback (Dietz et al., 1984, 1985b; Ackermann et al., 1986).

The P1 is followed by a larger negative potential with a peak in amplitude at 100–200 ms after the perturbation onset in the fronto-central cortical region, referred to as the N1 response. The N1 potential is the most pronounced and consistent feature of the PER (cf. Maki and McIlroy, 2007). In some earlier studies the N1 potential has been suggested to reflect cortical processing of sensory information flow induced by the perturbation (Dietz et al., 1984; Quant et al., 2004). However, the N1 response scales with perturbation amplitude (Staines et al., 2001; Mochizuki et al., 2010), is enhanced during higher postural threat (Adkin et al., 2008) and reduced or even absent when perturbation onset is predictable (Adkin et al., 2006). These results contradict the N1 potential to reflect solely the processing of afferent sensory input but rather they indicate the involvement of higher-order cognitive processes. Due to similarities in timing, shape, topography and spectral composition, the perturbation-evoked N1 response has been considered a functional analog of the “error negativity” (Ne; Falkenstein et al., 1991) or “error-related negativity” (ERN; Gehring et al., 1993) typically observed after an erroneous response during cognitive task performance. In the context of balance control, the term “error signal” has been introduced to describe the discrepancy between the expected and the actual state of balance after a sudden perturbation (Adkin et al., 2006, 2008; Maki and McIlroy, 2007; Mochizuki et al., 2010).

Similar to the Ne/ERN, the perturbation-evoked N1 appears at the midline with a fronto-central maximum which led to speculations about the possible involvement of the anterior cingulate cortex (ACC; cf. Maki and McIlroy, 2007). A recent source localization study (Marlin et al., 2014) revealed different dipole locations for the ERN while performing a flanker task and the N1 response following balance perturbations. While the dipole for the ERN was located in the cingulate gyrus (Brodmann area (BA) 24), the N1 dipole was located in the midline frontal gyrus, specifically the supplementary motor area (SMA, BA6). These results challenge the assumption the N1 is associated with the error signal processing in the ACC, and emphasize an important role of the SMA in controlling balance-recovery reactions. However, more research is needed to further unravel the functional properties and neuroanatomical substrates of the perturbation-evoked N1 response.

One characteristic pattern of results in a series of repetitive balance perturbations of the same kind is that the amplitude of postural reactions as well as muscular activation decreases over repeated trials (Horak and Nashner, 1986; Chong et al., 2000), and the largest amplitude reduction across trials typically occurs immediately between the first and the second trial (Oude Nijhuis et al., 2009, 2010; Pai et al., 2010; Nanhoe-Mahabier et al., 2012). In addition, Welch and Ting (2014) demonstrated a change in central sensitivity to kinematic errors induced by postural perturbations. Both kinematic error and muscle activity was found to decrease over repeated trials, however, the reduction in muscle activity was greater than that predicted by kinematic error alone, indicating a central change in sensorimotor processing of the muscle activity. Although such adaptation/habituation is a well-established phenomenon at the behavioral and neuromuscular level with regard to balance perturbations, it has not been identified yet at the cortical level. This can probably best be explained by the need to average multiple trials in order to achieve adequate signal-to-noise ratio (SNR) in the EEG measurement. However, as already mentioned above, the PER is a very robust finding and the large N1 component has already been successfully detected in single-trial responses to an unexpected perturbation (Adkin et al., 2006). Therefore, the PER may be well suited for single-trial analysis (Mouraux and Iannetti, 2008). The aim of this study was to apply single-trial analyses to a series of balance perturbations in a larger sample of young adult subjects in order to examine the P1 and N1 responses from trial to trial, and to identify the relationship between cortical responses and postural sway as well as activity of the plantar flexor muscles. In addition, P1 and N1 potentials were localized in order to examine whether the cortical locations remain consistent over successive perturbation trials.

It was hypothesized that postural sway and EMG activity of the ankle plantar flexor muscles will gradually decrease over trials indicating an adaptation. It should be noted that this would not be considered a true “motor adaptation” study in the motor learning community, where it is necessary to demonstrate aftereffects (Redding and Wallace, 2002) to show that the effects are due to stored central change. However, here those central changes are being examined directly. Due to its suggested sensory representation, the P1 potential was hypothesized to be located in the centro-parietal cortical region (specifically primary and secondary somatosensory cortex), and no changes in P1 amplitude and latency are expected over trials. The N1 response was hypothesized to be located in the fronto-central cortical region (specifically SMA). The N1 amplitude is expected to decrease over perturbation trials, indicating a decrease of fronto-central cortex activation as a result of experience and successful integration of “knowledge” about selective aspects of the perturbation characteristics such as direction and magnitude. However, the N1 response should not completely disappear as the timing of the perturbation remained unpredictable. Finally, it was hypothesized that the amplitude of the N1 potential, an index of cortical resources allocation to control balance-recovery reactions, is positively related to the amount of postural sway and plantarflexors EMG activity.

## Materials and Methods

### Participants

Thirty-nine healthy male university students participated in the study. Two subjects were excluded from subsequent data analyses due to technical problems during data recording resulting in excessive artifacts in the EEG and/or EMG signal. Therefore, the data of 37 participants (age: 24.7 ± 3 years; body weight: 77.3 ± 8.1 kg; height: 180.4 ± 5.1 cm; body mass index: 23.8 ± 2.4 kg/m^2^) were analyzed.

It has been shown that event-related potential (ERP) responses can significantly differ between females and males with regard to amplitude (e.g., Choi et al., 2015) and topography (e.g., Duregger et al., 2007). This suggests in ERP experiments results should not be generalized across sexes without prior statistical analysis, and both sexes should be included for a more comprehensive view. However, the single-trial approach in this study should benefit from a large sample size as averaging of the ERPs across a larger number of subjects is likely to improve the SNR. Therefore, within the scope of the possibilities available to us for this experiment, we decided to recruit a large sample of one sex only (i.e., males), being fully aware of the limited validity of our results for the other sex (i.e., females).

All subjects confirmed being free of injury for at least the last 6 months, having no pain or discomfort and/or experiencing any limitation in the range of motion during their daily routine and physical activity. In addition, all participants confirmed they did not undertake physical exercise in the 48 h prior to the experiment. Participants were informed about the experimental protocol and their written consent was obtained beforehand. The study was designed and performed according to the standards set by the declaration of Helsinki for medical research involving human subjects, and it was reviewed and approved by the local ethics committee of the German Sport University Cologne.

### Experimental Protocol

**Figure 1:**
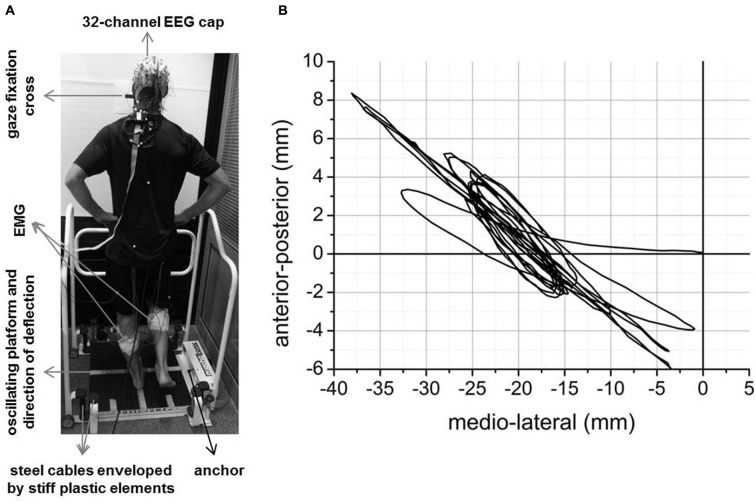
**Experimental setup (A) and an example of mean across trials platform oscillations during the initial 5 s after perturbation onset for a representative subject (B)**.

Figure 1A illustrates the experimental setup. Each participant completed ten trials of transient unpredictable balance perturbations using a Posturomed (Haider Bioswing, Pullenreuth, Germany). The number of trials was chosen based on previous research describing first-trial and adaptation effects with regard to balance-recovery reactions (Oude Nijhuis et al., 2009, 2010; Allum et al., 2011; Nanhoe-Mahabier et al., 2012) The Posturomed is a passively oscillating platform (60 × 60 cm) mounted to eight steel cables. The steel cables are enveloped by stiff plastic elements which “progressively dampen/attenuate” the oscillating behavior of the platform in response to external forces (i.e., muscular force). Progressively attenuated oscillating behavior means that the attenuation and thus, the deflection resistance is exponentially growing with increasing deflection of the platform. Platform oscillations of the Posturomed were recorded in anterior-posterior (*y*) and medial-lateral (*x*) direction using a non-contact inductive measurement system mounted underside. This system was calibrated before each trial. The corresponding software provides the *x* and *y*-coordinates of the platform with 100 Hz temporal and 0.1 mm spatial resolution. The absolute Euclidean distance (i.e., sway path length) in the time windows of interest was quantified. The mechanical constraints, validity and reliability of the Posturomed system have been described in several previous studies (Müller et al., 2004; Boeer et al., 2010a,b).

Each perturbation trial lasted 30 s in total. The trial started as soon as subjects confirmed that they were “ready” after positioning themselves on their dominant leg in center of the platform. Motor asymmetry was determined beforehand using the inventory of Reiss and Reiss (2000). Subjects were instructed to place their hands at the iliac crests, focus on a fixation cross at eye level, and following perturbation, keep platform oscillations to a minimum. The platform was laterally deflected by 2.2 cm and fixed by an anchor in this position. In each trial the investigator randomly released the anchor within the 5th and 20th second. Mean across subjects onset times did not significantly differ between trials (ANOVA: *F*_9,324_ = 0.38; *p* = 0.943). The release of the anchor induced a medial movement of the supporting platform. The inter-trial interval was set at 1 min to avoid fatigue. Perturbation onset timing was determined as the time point following anchor release at which the platform motion exceeded its mean oscillation level in medial-lateral or anterior-posterior direction during the last 5 s prior to anchor release by five standard deviations. An electrical pulse produced during anchor release was used to synchronize all measuring systems.

The rationale to use a medial-lateral perturbation was based on previous research reporting falling to the side accounts for a large proportion of falls (Maki and McIlroy, 1996), and lateral stability may be a better predictor of future falling risk than anterior-posterior stability (Maki et al., 1994). Single-limb stance was used based on the rationale that falling should occur predominantly during phases of single-limb support, as the base of support is much smaller compared to double-leg stance. Therefore, results obtained during single-limb stance may have greater ecological validity with respect to the risk of falls. We and others successfully recorded EEG during single-limb stance before (Slobounov et al., 2009; Hülsdünker et al., 2015), and Torres-Oviedo and Ting (2010) measured muscle activity in postural responses in single-limb stance vs. other stances.

### EEG and EMG Data Acquisition

EEG was recorded from 32 scalp locations (Brain Products GmbH, Munich, Germany) overlying the whole scalp and equally distributed over both hemispheres (FP_1/2_, F_7/8_, F_3/4_, F_*z*_, FC_5/6_, FC_1/2_, FC_*z*_, T_7/8_, C_3/4_, C_1/2_, C_*z*_, CP_5/6_, CP_1/2_, CP_*z*_, P_7/8_, P_3/4_, P_*z*_, O_1/2_, O_*z*_) according to the international 10:10-system (Jurcak et al., 2007). One additional electrode was used to measure electrooculographic (EOG) signals. The electrical reference and the ground electrode were located on position FCz and AFz, respectively. The sampling rate was set to 1000 Hz. Electrode impedances were kept below 5 kOhm.

EMG activity was recorded from the dominant (stance) and non-dominant (free) leg m. peroneus longus, m. gastrocnemius medialis and m gastrocnemius lateralis. DE-2.1 Ag single differential surface sensors (Bagnoli, Delsys, Natick, USA) with a contact spacing of 10 mm and an input impedance >10^15^ Ohm were used and amplified (1000×) by a Bagnoli^TM^ amplifier (Bagnoli, Delsys, Natick, USA). The skin was shaved, cleaned and abraded before sensors were attached using sensor-tailored adhesive interfaces. Data was sampled at 1000 Hz for offline analysis. Electrodes were placed on the muscle according to the SENIAM recommendations (SENIAM, 1999).

### EEG/EMG Data Processing and Analyses

EEG data were analyzed using the Brain Vision Analyzer 2 software (Brain Products, Munich, Germany). The data were first band pass filtered (Butterworth infinite impulse response, IIR) band-pass filter (2–30 Hz; 48 db/oct) and then segmented into epochs (trials) comprising the time interval −500 to 500 ms based on perturbation onset. Ocular artifacts were corrected using the Gratton and Coles ocular correction algorithm (Gratton et al., 1983). Cortical current density for each electrode position was calculated by Laplacian interpolation (order of splines: 4; Lambda: 1E^−005^). In each trial, the pre-perturbation baseline defined as the interval from −1000 to −500 ms relative to perturbation onset was subtracted from the data (baseline correction). The P1 peak was defined as the maximal positive voltage value compared to baseline in an interval from 0 to 100 ms following perturbation onset. The N1 peak was defined as the maximal negative voltage value compared to baseline in an interval from 100 to 300 ms following perturbation onset. P1/N1 latency was defined as the time interval between perturbation onset (time = 0) and the P1/N1 peak. Localization of P1 and N1 potentials was performed on a 20 ms time interval surrounding the peak positivity/negativity using the LORETA source localization (Pascual-Marqui et al., 1994) module integrated into Brain Vision Analyzer. EEG activity in the pre-perturbation time window (−500 to 0 ms, see below) was quantified as the band-pass filtered sum of activity values in the full spectrum (2–30 Hz).

The EMG data were first band-passed filtered (5–450 Hz; 48 db/oct) and then full-wave rectified. The segmentation and baseline correction procedure was analog to that described above for the EEG data. The magnitude of the EMG response is indicated as the integrated EMG activity.

**Figure 2:**
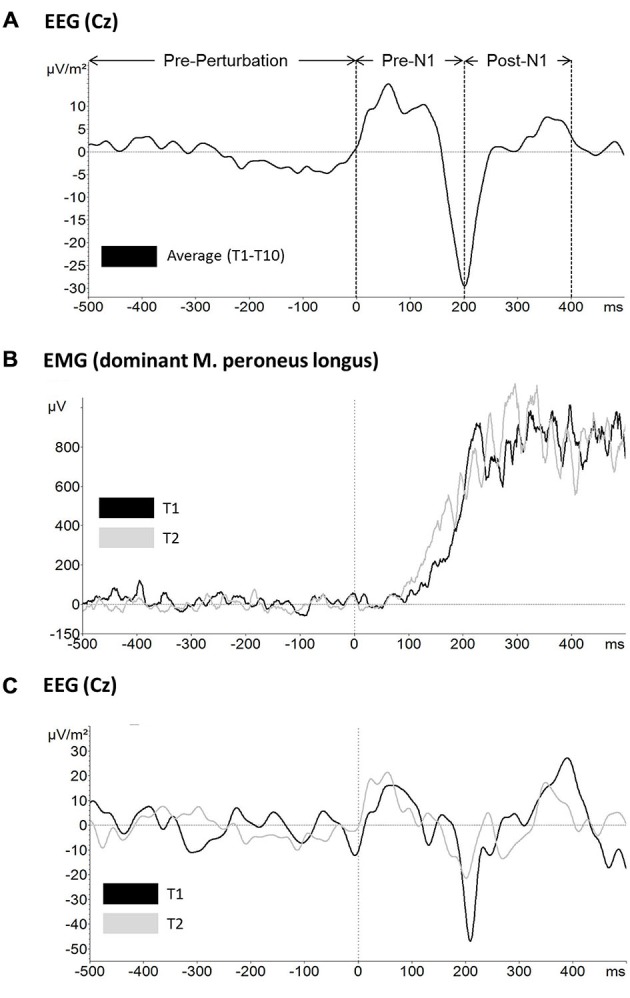
**Raw data traces for (A) mean across subjects and trials electroencephalographic (EEG) activity at electrode Cz and the time windows used for further analyses (top), (B) mean across subjects EMG activity of the dominant leg m. peroneus longus for trial 1 (black) and trial 2 (gray) and **(C)** mean across subjects EEG activity at electrode Cz for trial 1 (black) and trial 2 (gray)**.

Based on perturbation onset (time 0 ms) the following three time windows were defined for further analyses: −500 ms to 0 ms (pre-perturbation), 0–200 ms (pre-N1) and 200–400 ms (post-N1) (see Figure 2A). However, pre-perturbation platform oscillations were not included into analyses as the platform was rigid prior to perturbation onset (i.e., platform release). The rationale to use the peak of the N1 rather than the zero crossing before/after the N1 peak was based on the literature where the peak is typically used to determine the latency and amplitude of the N1 component (Adkin et al., 2006, 2008; Mochizuki et al., 2010).

### Statistics

All statistical analyses were conducted in Statistica 7.1 (StatSoft, Tusla, USA). Adaptation effects were analyzed using a one-way repeated measurement ANOVA with the within-subject factor TRIAL (T1–T10). The sphericity assumption was evaluated using the Mauchly’s test. The degrees of freedom were Greenhouse-Geisser corrected in case of non-sphericity. Significant TRAIL effects were further analyzed using the Fischer LSD *post hoc* test with the following significance levels: n.s. = not significant, *p < 0.05, **p < 0.01, ***p < 0.001. A significant difference between T1 and T2 for a given parameter was interpreted to indicate initial adaptation whereas longer-term adaptation should be indicated by a significant difference between T2 and T10 or, in case of absence of initial adaptation, between T1 and T10. In addition to the above described analyses, Pearson correlation coefficients were calculated to establish the relationship between EEG signal and behavior (i.e., platform oscillations) as well as EMG activity of the ankle plantarflexors in the time windows pre-N1 and post-N1, respectively. Prior to correlational analyses, each individual’s EEG and EMG values were *z*-transformed in order to account for interindividual differences in absolute values.

## Results

### Platform Oscillations

**Figure 3:**
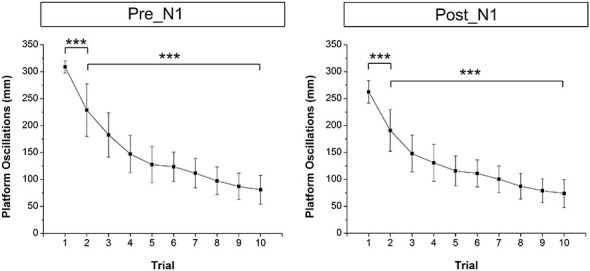
**Mean across subjects platform oscillations (i.e., Euclidean distance) for each trial in the time window pre-N1 and post-N1**. Platform oscillations were significantly lower in T1 compared to T2 (initial adaptation) as well as in T2 compared to T10 (longer-term adaptation). Error bars indicate 95% confidence intervals.

Figure 1B shows the mean across trials platform oscillations (i.e., Euclidean distance) of one subject during the initial 5 s after perturbation onset as an example. Mean across subjects platform oscillations in the time window pre-N1 and post-N1 for each trial are shown in Figure 3. The ANOVA yielded a significant TRIAL effect for both time windows (pre-N1: *F*_9,324_ = 47.7; *p* < 0.001; post-N1: *F*_9,324_ = 44.02; *p* < 0.001). *Post hoc* tests revealed a significant decrease in platform oscillations from T1 to T2 (pre-N1: *p* < 0.001; post-N1: *p* < 0.001) as well as from T2 to T10 (pre-N1: *p* < 0.001; post-N1: *p* < 0.001) indicating initial and longer-term adaptation, respectively.

### LORETA Source Localization of P1 and N1 Potentials

**Figure 4:**
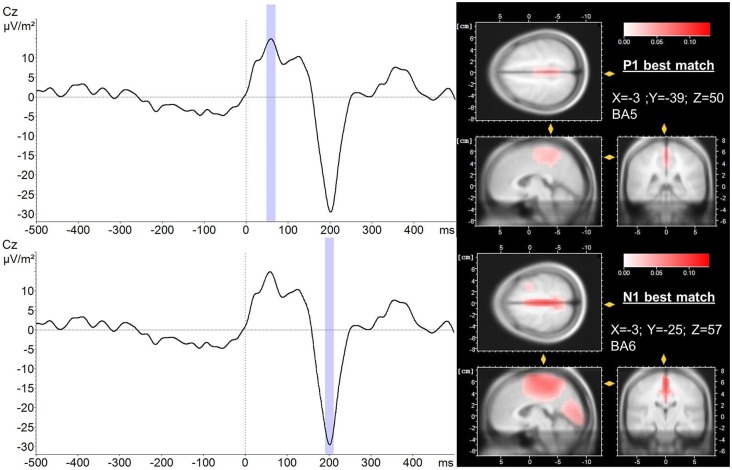
**LORETA localization (right) of the P1 (upper trace) and N1 (lower trace) potential averaged over trials and subjects (left)**. A 20 ms peri-peak window was considered for LORETA transformation (blue). LORETA anatomy slides are based on the MNI305 template and locked to the localization of maximal current density during the P1 and N1 potential, respectively. In addition, the coordinates of the voxel reflecting maximal current density (best match) and the corresponding Brodmann area (BA) are presented. Time 0 indicates perturbation onset.

LORETA source localization analysis revealed BA 5 and BA 6 as the dominant sources for the P1 and N1 potential, respectively. The average PER across trials and subjects as well as the corresponding localization of P1 and N1 potentials are presented in Figure 4. Best match cortical activity associated with the average P1 response (mean across subjects and trials) was located in BA5, a part of the posterior parietal cortex (PPC). The average N1 response (mean across subjects and trials) was located more anterior in the medial frontal gyrus (best match BA6) specifically the SMA. In contrast to the P1 potential, cortical activity associated with the N1 response was more distributed comprising BA24, a part of the ACC as well as BA5. In addition, Table 1 shows trial-by-trial best match LORETA coordinates for the P1 and N1 potential, respectively as well as the distance between the P1 and N1 best match locations. This overview indicates BA5 and BA6 were the best match locations for the P1 and N1 response, respectively in most of the trials. The mean across trials distance between the best match locations was 18.7 mm. This is greater than the average localization error of 9.2 ± 4.4 mm for sources at superior locations in the brain using spherical head models (Cuffin et al., 2001). However, the across trial standard deviation was 14.1 mm. This between-trial variation stems predominantly from variations in the *y*-coordinate, and it suggests the spatial resolution is probably too low to reliably differentiate between P1 and N1 locations between trials. However, it must be noted that the localizations of the across-subjects averaged P1 and N1 potentials during each trial are “weighted” for the individuals’ P1/N1 amplitude. That is, individuals with larger P1/N1 amplitude have a stronger effect on the average spatial distribution of cortical potentials which could bias the P1/N1 localization. Therefore, to test whether the chosen method provides sufficient spatial resolution to differentiate between the P1 and the N1 location in general (i.e., across trials and subjects) we submitted the individual LORETA *x*, *y* and *z*-coordinates of the across-trial average P1 and N1 potentials to an ANOVA with the factors POTENTIAL (P1 vs. N1) and COORDINATE (*x*, *y* and *z*). This ANOVA yielded a significant POTENTIAL × COORDINATE interaction (*F*_2,72_ = 9.25; *p* < 0.001). *Post hoc* tests revealed the LORETA coordinates of the P1 and N1 potentials were significantly different with the P1 potential located posterior (*p* < 0.001) and slightly more left-sided (*p* = 0.049) to the location of the N1 potential. Hence, the cumulative pattern of these results suggests the localization method provides sufficient spatial resolution to distinguish between the P1 and N1 locations when using the individuals’ across-trial averaged P1/N1 potentials but it fails to reliably differentiate between P1 and N1 locations between trials.

**Table 1:** **Trial-by-trial and mean best match LORETA coordinates and the corresponding Broadmann area (BA) for the P1 and N1 potential, respectively**.

	P1				N1				Distance P1-N1
Trial	*X*	*Y*	*Z*	*BA*	*X*	*Y*	*Z*	*BA*	mm
T1	−3	−11	64	6	−3	−11	64	6	0.0
T2	−3	−39	50	5	−3	−11	64	6	31.3
T3	−3	−39	50	5	−3	−25	57	6	15.7
T4	−3	−39	50	5	−3	−11	64	6	31.3
T5	−3	−39	50	5	−3	−11	64	6	31.3
T6	−3	−46	57	5	−3	−39	57	5	7.0
T7	−3	−11	64	6	−3	−11	64	6	0.0
T8	4	−39	50	7	−3	−11	64	6	32.1
T9	−3	−39	50	5	−3	−11	64	6	31.3
T10	−3	−46	50	7	−3	−46	57	5	7.0
Mean (T1–T10)	−2.3	−34.8	53.5		−3	−18.7	61.9		18.7
SD	2.2	12.9	5.9		0	13.4	3.4		14.1

*Last column indicates the distance between the best match locations of the P1 and N1 potentials*.

### P1 and N1 Amplitude/Latency

**Figure 5:**
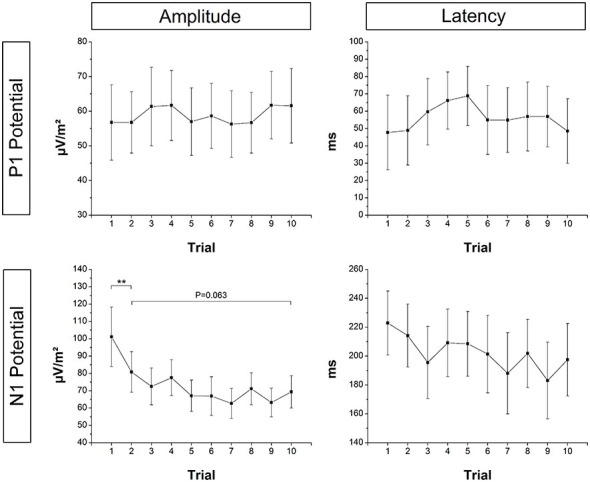
**Mean across subjects P1 and N1 amplitude and latency at electrode CPz and FCz, respectively**. N1 amplitude was significantly smaller in T1 compared to T2 (initial adaptation), and there was a strong trend towards a further reduction of the N1 amplitude from T2 to T10 (longer-term adaptation). Error bars indicate 95% confidence intervals.

Mean EEG raw data traces at electrode Cz are shown in Figure 2. Based on the above described LORETA results, FCz and CPz were chosen as representative electrodes for analysis of the N1 and P1 potential, respectively. Mean across subjects P1 and N1 amplitude/latency for each trial are shown in Figure 5, respectively. The ANOVA yielded a significant TRIAL effect for the N1 amplitude (*F*_9,324_ = 6.80; *p* < 0.001). *Post hoc* testing revealed N1 amplitude was significantly larger in T1 compared to T2 (*p* = 0.001) indicating initial adaptation. In addition, a clear trend towards longer-term adaptation was observed in the subsequent trials (T2 vs. T10, *p* = 0.063). No significant TRIAL effects were found for N1 latency (*F*_9,324_ = 1.12; *p* = 0.349), P1 amplitude (*F*_9,324_ = 0.38; *p* = 0.946) and P1 latency (*F*_9,324_ = 0.69; *p* = 0.721).

### Ankle Plantarflexors EMG Activity

**Figure 6:**
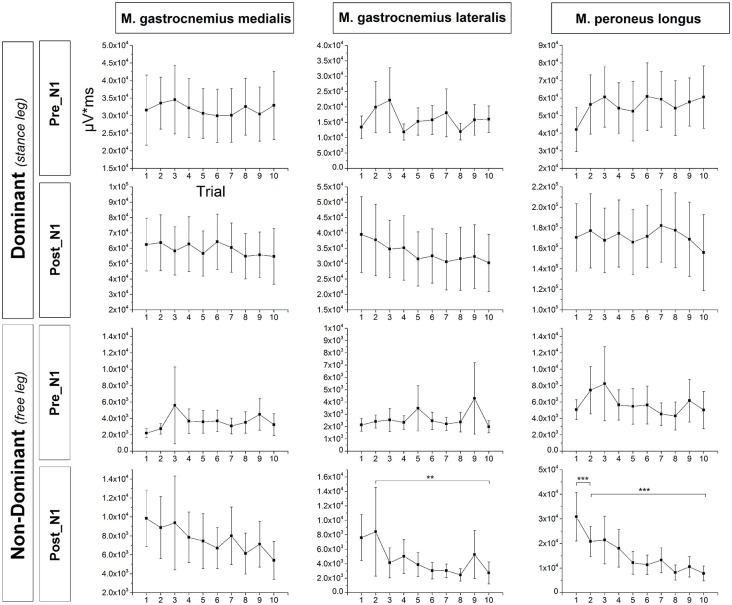
**Mean across subjects integrated EMG activity of the ankle plantarflexors in the time window pre-N1 and post-N1 for each trial**. EMG activity of the non-dominant m. gastrocnemius lateralis in the time window post-N1 was significantly larger in T1 compared to T10 but not in T1 compared to T2 indicating longer-term but not initial adaptation. Furthermore, EMG activity of the non-dominant m. peroneus longus in the time window post-N1 was significantly larger in T1 compared to T2 as well as in T2 compared to T10 indicating both initial and longer-term adaptation. Error bars indicate 95% confidence intervals.

Exemplar EMG raw data traces for the m. peroneus longus of the dominant (stance) leg are shown in Figure 2B. Mean across subjects integrated EMG activity of the ankle plantarflexors for each trial is shown in Figure 6. The ANOVA yielded a significant TRIAL effect for the m. gastrocnemius lateralis (*F*_9,324_ = 3.04; *p* = 0.042) and m. peroneus longus (*F*_9,324_ = 12.19; *p* < 0.001) of the non-dominant (free) leg in the time window post-N1. No further TRIAL effects were observed for any of the other muscles (*pre-N1 dominant leg*: m. gastrocnemius medialis: *F*_9,324_ = 0.62; *p* = 0.728; m. gastrocnemius lateralis: *F*_9,324_ = 2.07; *p* = 0.107; m. peroneus longus: *F*_9,324_ = 1.09; *p* = 0.367; *pre-N1 non-dominant leg*: m. gastrocnemius medialis: *F*_9,324_ = 1.16; *p* = 0.320; m. gastrocnemius lateralis: *F*_9,324_ = 1.79; *p* = 0.177; m. peroneus longus: *F*_9,324_ = 1.31; *p* = 0.269; *post-N1 dominant leg*: m. gastrocnemius medialis: *F*_9,324_ = 1.03; *p* = 0.403; m. gastrocnemius lateralis: *F*_9,324_ = 1.96; *p* = 0.129; m. peroneus longus: *F*_9,324_ = 1.16; *p* = 0.328; *post-N1 non-dominant leg*: m. gastrocnemius medialis: *F*_9,324_ = 1.61; *p* = 0.167).

*Post hoc* testing revealed EMG activity of the non-dominant m. gastrocnemius lateralis in the time window post-N1 was significantly larger in T1 compared to T10 (*p* = 0.004) but not in T1 compared to T2 (*p* = 0.629) indicating longer-term but not initial adaptation. Furthermore, EMG activity of the non-dominant m. peroneus longus in the time window post-N1 was significantly larger in T1 compared to T2 (*p* = 0.001) and T2 compared to T10 (*p* < 0.001) indicating both initial and longer-term adaptation.

### Pre-Perturbation EEG and EMG Activity

There were no significant changes in pre-perturbation (−500 to 0 ms) EEG (*F*_9,324_ = 1.02; *p* = 0.426) and EMG (*dominant leg*: m. gastrocnemius medialis: *F*_2,72_ = 0.49; *p* = 0.614; m. gastrocnemius lateralis: *F*_2,72_ = 0.01; *p* = 0.991; m. peroneus longus: *F*_2,72_ = 0.41; *p* = 0.663; *non-dominant leg*: m. gastrocnemius medialis: *F*_2,72_ = 1.41; *p* = 0.250; m. gastrocnemius lateralis: *F*_2,72_ = 3.58; *p* = 0.054; m. peroneus longus: *F*_2,72_ = 1.58; *p* = 0.213) activity over trails.

### Correlational Analyses

**Figure 7:**
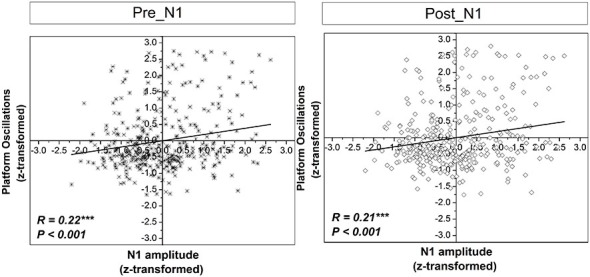
**Correlations between *z*-scores of the standard normal distribution for the N1 amplitude and platform oscillations (i.e., Euclidean distance) across all subjects and trials in the time window pre-N1 and post-N1**. *R* and *p*-values indicate correlation coefficients and significance level, respectively.

**Figure 8:**
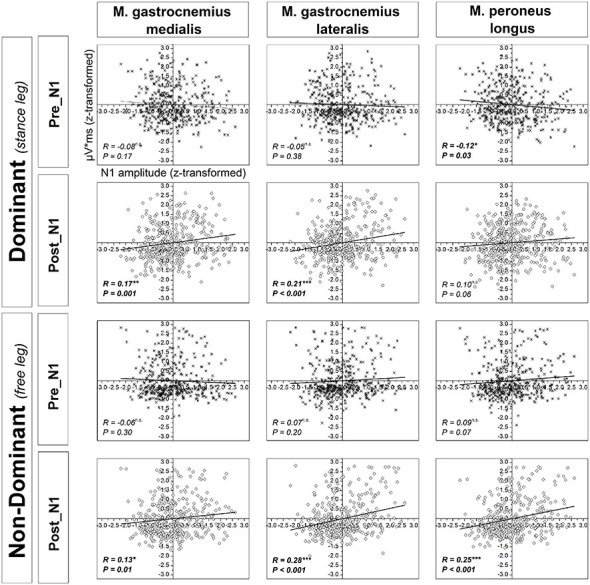
**Correlations between *z*-scores of the standard normal distribution for the N1 amplitude and muscular activity of the ankle plantarflexors across all subjects and trials in the time window pre-N1 and post-N1**. *R* and *p*-values indicate correlation coefficients and significance level, respectively. Significant *R/P*-values are presented in bold.

Significant positive correlations were found between *z*-scores of N1 amplitude and the *z*-scores of platform oscillations for both time windows after perturbation onset (Figure 7). In addition, a weak but statistically significant negative correlation was found between the *z*-scores of N1 amplitude and the *z*-scores of EMG activity only for the dominant m. peroneus longus in the time window pre-N1. In contrast, in the time window post-N1, significant positive correlations were found between the *z*-scores of N1 amplitude and the *z*-scores EMG activity of all examined plantar flexor muscles, except for the dominant m. peroneus longus (Figure 8).

## Discussion

This study aimed to identify cortical characteristics associated with adaptive behavior during a series of repetitive balance perturbations unpredictable in timing. To this end, P1 and N1 perturbation-evoked potentials were analyzed trial by trial in young healthy men, and correlated with individuals’ behavioral and plantarflexors EMG responses. In addition, LORETA source localization analysis was applied in order to identify the cortical sources of the perturbation-evoked P1 and N1 responses, and to evaluate the consistency of the localizations over trials.

### Behavioral Data and Ankle Plantarflexors EMG Activity

As expected, platform oscillations decreased over trials indicating that participants’ behavior adapted to the demands of the task. However, the EMG activity of the dominant (stance) leg plantarflexors remained unchanged over trials. At a first glance this counters prior adaptation studies (Horak and Nashner, 1986; Chong et al., 2000; Welch and Ting, 2014) however, it appears plausible when one considers that the subjects were instructed “to keep platform oscillation to a minimum” which is theoretically zero. Consequently, as long as the platform oscillates, the plantarflexors of the balancing leg should remain activated to a high degree in order to stop the platform from oscillating. In contrast to the dominant leg, in the non-dominant (free) leg m. gastrocnemius lateralis and m. peroneus longus a significant reduction of EMG activity over trials was found in the time window post-N1. One possible explanation for this finding could be that the degree of co-contraction decreases over successive trials as a result of selective neuromuscular activation (i.e., improved coordination). The fact that no such adaptation was observed in the time window pre-N1 suggests the reduction of co-contraction is probably organized at the cortical level. However, this is needs to be further studied as Welch and Ting (2014) showed reduced co-contraction during repeated perturbations to standing, and the latencies were fast enough to be brainstem mediated (i.e., after about 100 ms). However, this has been demonstrated for antagonist-agonist co-contraction, and not for muscles of the contralateral (free) leg as observed in this study. Specifically, in the former case, the antagonist-agonist co-contraction may play a functional role whereas in the latter case it is probably a (adverse) concomitant. Also, the balance tasks differed substantially between studies.

In addition to co-contraction, non-dominant leg plantarflexors activation may also result from temporary bipedal stance to avoid falls. Such compensatory reactions would be expected to occur predominantly in the “late-phase” (i.e., post-N1) of the balance-recovery (Chvatal et al., 2011; Potocanac et al., 2014), and at the beginning of the perturbation series. However, as we did not explicitly record the amount of temporary bipedal stance in the analyzed time windows this explanation remains speculative.

### Localization and Trial Effects of the P1 and N1 Response

While the P1 potential remained unchanged in amplitude and latency over trials, the amplitude of the N1 potential was significantly reduced in the second compared to the first trial indicating a pronounced initial adaptation. Furthermore, N1 amplitude showed a clear trend towards a further decrease in the subsequent trials indicating longer-term adaptation. The P1 potential is suggested to reflect the first cortical representation of sensory afferents arising from the periphery following a perturbation (Dietz et al., 1984; Ackermann et al., 1986; Duckrow et al., 1999; Adkin et al., 2006). The results of the present study further support this hypothesis. Although platform oscillations following the perturbation were reduced over trials, the initial perturbation magnitude was kept constant. Accordingly, the initial sensory discharge from the periphery is suggested to remain unchanged, causing a consistent P1 response over trials. Further support for the peripheral origin of the P1 potential comes from the LORETA source localization analysis. The primary source of cortical P1 activity was located in BA5, a part of the PPC which receives and integrates somatosensory input from the periphery (Andersen et al., 1997).

In contrast to the P1 response, the N1 potential has been shown to be affected by perturbation amplitude (Staines et al., 2001; Mochizuki et al., 2010), perturbation predictability (Adkin et al., 2006), postural threat (Adkin et al., 2008) and performance of a concurrent cognitive task (Quant et al., 2004). These results indicate the N1 response does not reflect pure sensory processing as previously suggested (Dietz et al., 1984, 1985b) but rather it is also associated with higher order cognitive processes. Adkin et al. (2006) demonstrated that although perturbation amplitude was constant, N1 amplitude was markedly reduced or even absent following predictable when compared to unpredictable perturbations. The authors suggested these results to indicate a reduction of the discrepancy between the expected and actual state of balance (error signal) in the predictable trials. In this context, the reduction of the N1 amplitude over trials found in this study may be attributable to changes in cortical activation associated with error signal processing as the discrepancy between the expected and actual state of balance is expected to decrease with additional knowledge about the amplitude and direction of the perturbation (Mochizuki et al., 2010). This interpretation is in agreement with the results of Horak et al. (1989) demonstrating prior experience as well as knowledge of perturbation amplitude reduced the compensatory reaction. Furthermore, anticipation of perturbation onset has been shown to not only induce post-perturbation changes in N1 amplitude, but also adaptations in pre-perturbation cortical and muscular activity (Jacobs et al., 2008; Mochizuki et al., 2008; Slobounov et al., 2013). To exclude that the latter was the case in our study; we have also analyzed EEG and EMG activity prior to perturbation onset for each trial, respectively. Neither pre-perturbation EEG nor EMG activity changed over trials suggesting the participants were not able to anticipate the onset of the perturbation. However, it should be noted that Welch and Ting (2014) found a change in pre-perturbation m. gastrocnemius medialis EMG activity associated with predicting perturbation direction (i.e., forward/backward). One reason we did not found such an effect in our study could be the difference between studies with regard to the postural configuration. Specifically, the change in pre-perturbation EMG activity found by Welch and Ting (2014) during double-leg stance was associated with a coherent shift of the initial body lean. Shifts of the center of mass produced in the ankle are likely to be more pronounced during double-leg stance compared to single-leg stance due to the larger base of support. In addition, because of the anatomy of the ankle joint, the margin for a center of mass shift produced in the ankle is much smaller in medial-lateral direction compared to anterior-posterior direction.

It has been speculated the ACC is the primary neuroanatomical substrate for error signal processing during balance control (cf. Maki and McIlroy, 2007). However, in the present study, although the N1 source localization analysis revealed some activity in the ACC (BA 24), the best match coordinates corresponded to the medial frontal gyrus (BA 6), specifically the SMA. This result emphasizes the importance of the SMA in controlling balance-recovery reactions, and it further supports the results of Marlin et al. (2014) suggesting the N1 potential is generated in the SMA, not the ACC. A decrease of SMA activation over trials, as revealed by reduced N1 amplitude, indicates the SMA is particularly important for performance of demanding balance tasks (i.e., when performance is less automatic). This is further supported by a very recent functional magnetic resonance imaging study demonstrating activity in the SMA (and cerebellum) was higher during motor imagery of a more demanding balance task (Taube et al., 2015). Although our results with regard to the localization of the N1 response are generally in agreement with a previous study by Marlin et al. (2014), they should be interpreted with caution. Specifically, sources identified using a larger number of electrodes and projected onto a magnetic resonance image of the individuals’ brain will improve the exact localization of the EEG signal (Michel and Murray, 2012). Furthermore, although for the purpose of this study single-trial EEG analysis is useful, a critical discussion of this approach is warranted. Specifically, for smaller amplitude ERPs there is a high risk of spurious detection of uncorrelated noise resembling the searched-for visual template. This can make replication of results difficult even if an independent observer is assigned to detect the ERPs. In addition, the ERP component in coincidence with noise may lead to an overestimation of the response amplitude (Mouraux and Iannetti, 2008). However, despite these limitations, manual measurement of single-trial ERPs has been shown to work reasonably well for components with larger amplitudes (Iannetti et al., 2005), and it is less prone to a bias due to variability in latency (Purves and Boyd, 1993). Similar to previous studies, in this study the N1 represents a relatively large amplitude signal resulting in very good SNR. This, and the relatively large number of subjects, are likely to facilitate the localization of the PER, and make identification of the response in single trials straightforward.

We found no indication for a relocation of the source of the N1 response over repeated trials suggesting the SMA remains the dominant cortical source for the task applied in this study independent of a reduction of the N1 amplitude and behavioral adaptations. However, this is a hypothesis to be challenged in future studies rather than a final conclusion as the localization method failed to provide sufficient spatial resolution to reliably differentiate between the location of the P1 and N1.

### Relationship Between Cortical Activity and Behavior/Muscular Activity

It was found that early activation of the m. peroneus longus, a muscle that is crucially involved in postural control of single-limb stance (Tropp and Odenrick, 1988), is associated with a reduction of cortical resources allocated to control “late-phase” (i.e., post-N1) balance-recovery reactions. Specifically, the amount of cortical resources allocated to control balance-recovery reactions, as reflected by the N1 amplitude, is larger when the initial activation of the m. peroneus is lower. This suggests early activation of the dominant m. peroneus longus is an important mechanism for stability. The initial stability, in turn, is likely to be the reason for reduced allocation of cortical resources associated with preparation of late-phase motor responses.

In contrast to the above discussed results with regard to the time window pre-N1, in the time window post-N1, significant positive correlations were obtained between the N1 amplitude and EMG activity of all analyzed muscles, except for the dominant m. peroneus longus. This pattern of results suggests stronger cortical activation is associated with a higher level of subsequent co-contraction of the ankle plantarflexors. In addition, the correlations between non-dominant leg plantarflexors EMG activity and N1 amplitude could be partially caused by near-falls when subjects are temporarily forced to bipedal stance and thus, exhibit increased EMG activity in the non-dominant leg. The fact that this relationship is weak and not significant for the dominant m. peroneus longus may be attributed to a very high activation of the dominant leg plantarflexors throughout trials due to task demands (cf. 4.1). Apart from that, it is well-established the neuromuscular activation strategies to control balance-recovery reactions are complex, and typically include not only ankle plantar flexor muscles but also muscles of the knee, hip, trunk, neck and shoulders (Oude Nijhuis et al., 2009; Welch and Ting, 2014). Furthermore, neuromuscular activation strategies are highly individual, and are likely to change over trials (Torres-Oviedo and Ting, 2010; Welch and Ting, 2014) which may also contribute to a reduction of the correlational strength between cortical activity and activation of single muscles. Finally, it should be noted that the change in N1 amplitude over trials should not necessarily be related to EMG magnitude. Prior work demonstrated that anticipated perturbations elicit no N1 response, despite postural responses (Adkin et al., 2006). This suggests sub-cortical processing of EMG responses parallel to the cortical response.

## Conclusion

This is the first study to identify adaptation effects at the cortical level in the context of balance-recovery reactions. The results emphasize the essential information, single-trial analyses of PERs can provide to the current knowledge on cortical control of balance. The reported adaptation effects for the N1 potential and the observed correlations between N1 amplitude and postural sway provide further insights into cortical processes associated with higher fall incidence rates commonly reported during initial presentation of a balance disturbance. In addition, LORETA source localization of the P1 and N1 potentials indicates an important role of the PPC (P1 potential) and SMA (N1 potential) in balance control.

## Funding

This study was supported by the DFG (INST 229/2-1 FUGG). TH is a recipient of the graduate school doctoral scholarship at the German Sport University Cologne.

## Conflict of Interest Statement

The authors declare that the research was conducted in the absence of any commercial or financial relationships that could be construed as a potential conflict of interest.
